# Microleakage of Restorative Materials Used for Temporization of Endodontic Access Cavities

**DOI:** 10.3390/jcm12144762

**Published:** 2023-07-18

**Authors:** Sabina Noreen Wuersching, Luise Moser, Katharina Theresa Obermeier, Maximilian Kollmuss

**Affiliations:** 1 Department of Conservative Dentistry and Periodontology, LMU University Hospital, LMU Munich, 80336 Munich, Germany; sabina.wuersching@med.uni-muenchen.de (S.N.W.);; 2 Department of Oral and Maxillofacial Surgery and Facial Plastic Surgery, LMU University Hospital, LMU Munich, 80337 Munich, Germany; katharina.obermeier@med.uni-muenchen.de

**Keywords:** microleakage, bacterial penetration, glucose leakage, temporary restorative materials

## Abstract

A tight temporary seal applied to an access cavity is thought to improve endodontic outcomes. This study aims to assess the bacterial and glucose microleakage of different types and combinations of temporary restorations. Human-extracted incisors were instrumented, dressed with a calcium hydroxide paste, and sealed with Cavit W (CW), CW/Ketac Molar (CW/KM), CW/Smart Dentin Replacement (CW/SDR), Intermediate restorative material/KM (IRM/KM), or Clip F (CF). Standardized 3D-printed hollow test specimens were manufactured and temporized in the same manner. The specimens were examined for bacterial and glucose leakage for 28 days. Data were analyzed using a Kaplan–Meier survival analysis. CW/SDR and CF showed the least bacterial and glucose leakage over time. CW, CW/KM, and IRM/KM had similarly high levels of glucose leakage, but CW/KM and IRM/KM provided a tighter seal against bacterial penetration than CW. CW/SDR and CF should be considered for the sealing of access cavities of teeth previously restored with methacrylate-based materials.

## 1. Introduction

The persistence of bacteria in the root canal is one of the major causes of recurrent endodontic infections [1]. Several steps during endodontic treatment, such as mechanical instrumentation, antiseptic control with irrigation solutions, the insertion of dressing agents with antibacterial properties, and the use of a rubber dam, are intended to eradicate bacteria from the root canal or prevent bacterial recolonization [2]. A further strategy for improving endodontic outcomes is an adequate temporary seal of the access cavity, which prevents the contamination of the root canal system with oral fluids and bacteria and forestalls the seepage of intracanal medicaments between appointments [3]. The significance of a permanent coronal seal after obturation with respect to long-term success has been previously demonstrated [4]. However, the coronal microleakage of temporary restorations must also be considered a potential etiological agent for persistent endodontic infections. In fact, unsatisfactory temporary restorations have been identified as one of the major factors associated with continuing pain after root canal preparation [5]. Aside from resistance to microleakage being the main requirement for temporary coronal restorations, there are further criteria that should be considered when choosing a temporary restorative material for endodontic treatment. From a practical point of view, the material should have a simple application procedure, but it should also be easy to remove, leaving virtually no residue in the access cavity. Furthermore, cost effectiveness is an important consideration for clinicians when selecting any dental material for routine use in clinical practice, specifically in endodontic cases requiring multiple temporary seals.

Among the wide selection of available restorative materials, Cavit has been the most popular temporary material in endodontics for many decades. This popularity is mainly attributed to its simple application procedure, quick removal, and low cost. Cavit is a eugenol-free calcium sulphate-zinc oxide-based cement that has self-curing properties in the presence of moisture. The setting reaction of Cavit is initiated by water, which reacts with calcium sulfate and zinc-oxide-sulfate and thereby produces a hard cement. The coronal sealing ability of Cavit have been studied previously; however, the in vitro results seem to vary depending on the method used for assessing the sealing ability [6]. In a recent systematic review comparing the coronal leakage of different orifice barriers, Cavit showed superior bacterial microleakage values compared to the other tested materials [7].

To enhance the coronal sealing ability of Cavit, it is a common practice to apply an additional material to the access cavity on top of a layer of Cavit [8]. For this purpose, flowable composites or glass ionomer cements (GICs) have been suggested. Both material classes were primarily developed for restoring class I and II cavities, where a tight marginal seal is required for the prevention of secondary caries. This makes composites and GICs attractive for temporization during endodontic treatment. However, composites require an additional step for a sufficient seal, that is, enamel and dentin conditioning with phosphoric acid and a dentin-bonding agent. Since composites and GICs originated in restorative dentistry, they were developed with the premise of being able to last for longer periods of time, which makes the removal of these materials from an access cavity potentially more difficult compared to Cavit. Light-curing bulk fill restorative materials such as Clip F allow for easy placement and removal and should, therefore, be considered for the temporary sealing of endodontic access cavities. Clip F is a methacrylate-based restorative material with fluoride-releasing properties that bonds to a tooth without requiring any pretreatment of the cavity. Its alleged expansion features are thought to ensure a tight seal of a restoration; however, only few studies have examined the sealing ability of this material so far [9,10].

Another material that is frequently used for temporization in endodontics is IRM Intermediate restorative material. IRM is a polymethyl methacrylate reinforced zinc oxide-eugenol cement that was developed for all kinds of intermediate restorations that are intended to remain in place for up to a year. The eugenol content provides pain relief and is thus frequently applied to teeth with hypersensitive pulps or as a pulp-capping material in primary teeth [11]. However, there are conflicting findings from in vitro and in vivo studies regarding the sealing ability of IRM, with some studies showing equal or more favorable results than Cavit and other studies demonstrating inferior sealing properties compared to Cavit [6]. Adding a layer of GIC on top of IRM has been shown to improve the marginal seal and prevent leakage over a period of up to one month [12].

Although the tightness of some of these materials has been previously examined, there are only few data on the bacterial leakage of combined restorative materials. Therefore, the main objective of this study is to assess the sealing ability of different types and combinations of restorative materials used for the temporization of endodontic access cavities.

## 2. Materials and Methods

### 2.1. Temporary Filling Materials

Five different combinations of temporary restorative materials were examined in this study: Cavit W (CW, 3M, St. Paul, MN, USA), Cavit W/Ketac Molar Aplicap (CW/KM, 3M), Cavit W/Smart Dentin Replacement (CW/SDR, Dentsply Sirona, York, PA, USA), IRM (Dentsply Sirona)/Ketac Molar Aplicap (IRM/KM), and Clip F (CF, Voco Dental GmbH, Cuxhaven, Germany). Table 1 shows an overview of all materials used in this study along with the detailed application procedures used for the corresponding restorations.

### 2.2. Bacterial Strains

All bacterial strains were obtained from the German Collection of Microorganisms and Cell Cultures (DSMZ, Braunschweig, Germany). The following bacteria were used in this study: *Streptococcus mutans* (DSM 20523), *Aggregatibacter actinomycetemcomitans* (DSM 11123), and methicillin-resistant *Staphylococcus aureus* (MRSA, DSM 11822). All strains were grown and maintained on Schaedler agar plates supplemented with vitamin K1 and 5% sheep blood (Becton Dickinson, Franklin Lakes, NJ, USA). For growth in liquid media, the bacteria were cultured in Brain Heart Infusion Broth (BHI, Becton Dickinson) supplemented with hemin (5 µg/mL) and vitamin K1 (1 µg/mL). The bacteria were incubated at a temperature of 37 °C and a humidity level of 60% in a CO_2_-enriched atmosphere with 5.8% CO_2_.

### 2.3. Determination of Antibiotic Susceptibility Profiles

The bacterial penetration experiment required a BHI medium supplemented with antibiotics to avoid cross-contamination and prevent growth of typical pathogens that may originate from extracted teeth. The antibiotic susceptibility of *S. mutans* and *A. actinomycetemcomitans* to ampicillin (AMP) and ciprofloxacin (CIP) was determined using the ETEST method (bioMèrieux SA, Marcy-l’Étoile, France). In brief, colonies of *S. mutans*, *A. actinomycetemcomitans* and MRSA were inoculated in BHI and adjusted to a McFarland Standard of 0.5. The bacterial suspensions were evenly streaked onto individual Schaedler agar plates using sterile cotton swabs. The agar plates were incubated with ETEST strips under the required growth conditions for 24 h, and minimum inhibitory concentrations (MICs) were read and interpreted according to the breakpoints reported in the guidelines from the Clinical and Laboratory Standards Institute (CLSI) [13,14]. All tests were performed in triplicate for each bacterial strain. Based on the MICs, antibiotic concentrations were selected in order to supplement the BHI medium with AMP and CIP. The prepared BHI was examined in terms of the selective bacterial growth of MRSA by plating and culturing bacterial suspensions of all three strains grown in BHI supplemented with the antibiotics.

### 2.4. Preparation of Human-Extracted Teeth

To test the tightness of the temporary restorative materials, a previously described method was used and modified [15,16]. The use of extracted human teeth was approved by the local ethics committee (registration No. 23-0431 KB). A total of 75 extracted, single-rooted, anterior human teeth with mature apices and no signs of caries in the root area were selected and X-rayed. The coronal portion of all teeth was shortened with a diamond saw to standardize the teeth to a working length of 16 mm. The access opening of each tooth was also standardized (5 mm depth, 2.5 mm diameter). The coronal third of the root canal was flared with Gates–Glidden burs, and root canal preparation was performed with a reciprocating single file (Reciproc blue R40, VDW, Munich, Germany). Each root canal was irrigated with 15 mL of 3% sodium hypochlorite (NaOCl) and 5 mL of 20% ethylenediaminetetraacetic acid (EDTA), and the teeth were then sterilized at 121 °C for 15 min. The apical 7 mm sections of the root canal were filled with a calcium hydroxide paste (Ultracal XS, Ultradent Products, South Jordan, UT, USA) using a 28 G (0.35 mm) needle (VMK Endoneedle, VMK, Dilbeek, Belgium). The teeth were divided into five experimental groups (n = 15) to seal the opening of the access with the different restorative materials. Detailed specifications of the filling procedure for each material are shown in Table 1.

### 2.5. Preparation of 3D-Printed Hollow Test Specimens

Since the inherent anatomical variations of the extracted teeth can affect the corresponding results, standardized test specimens that simulate an access cavity in a resin-based material were created. A hollow, conical cylinder measuring 8 mm in height and with an inner diameter of 2.5 mm at the frustum base corresponding to the dimensions of the access openings of the extracted teeth was virtually designed using CAD software (Tizian Creativ RT-Software, Version 3.1, Schütz Dental GmbH, Rosbach, Germany). A diagram showing the dimensions of the conical cylinder is displayed in the Supplementary Material (Figure S1). The template was exported as a standard tessellation language (stl) file and sent to a 3D-printer (Form 2, Formlabs Inc., Somerville, MA, USA), where 150 specimens were produced from a printable resin material (Dental LT Clear Resin V2, Formlabs). The resin specimens were post-processed according to the manufacturer’s instructions by washing them in isopropanol for 20 min (Form Wash, Formlabs) and post-curing them at 60 °C for 60 min (Form Cure, Formlabs). The hollow test specimens were filled with the restorative materials in the same manner as described in Table 1.

### 2.6. Bacterial Penetration

**Figure 1:**
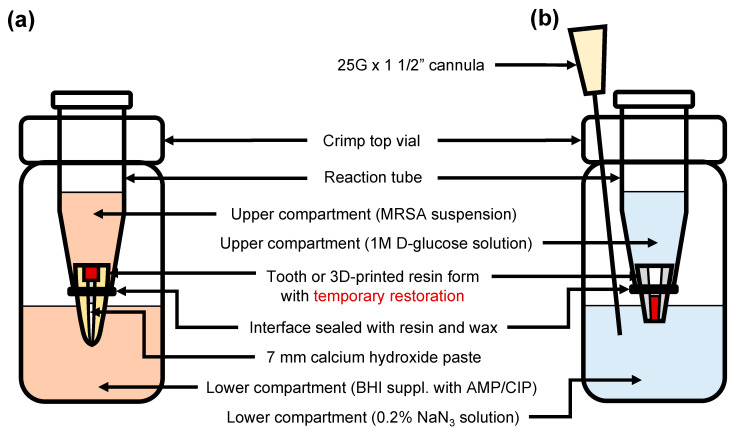
Diagram showing the experimental setup for assessing (**a**) bacterial penetration and (**b**) glucose leakage.

Each filled tooth and hollow test specimen was placed into an individual reaction tube that was cut off at the bottom to expose the tooth apex/small frustum base. The interface between the tooth/test specimen and the reaction tube was sealed with light-curing flowable resin (SDR flow+, Dentsply Sirona Deutschland GmbH, Bensheim, Germany) and wax. A hole measuring the same diameter of the reaction tube was drilled into the cap of a sterile crimp top vial. The reaction tube with the sealed specimen was placed into the hole, thereby creating two compartments with the filled tooth apex/hollow test specimen as the only interface. The experimental setup is shown in Figure 1a. MRSA was inoculated in BHI medium supplemented with antibiotics (10 µg/mL AMP; 10 µg/mL CIP), and 500 µL of the MRSA suspension was added to the reaction tubes (upper compartment). The crimp top vial (lower compartment) was filled with clear BHI medium supplemented with antibiotics, enough to cover 2 mm of the tooth apex/hollow test specimen in liquid. This setup was incubated for 28 days at 37 °C in a humidified atmosphere containing 5% CO_2_. Once every four days, the bacterial suspension in the upper compartment was replaced with a fresh MRSA suspension. The medium in the lower compartment was examined for turbidity, as a sign of bacterial growth, every day. In case the medium of a specimen turned turbid, the day of the event was recorded, and the specimen was not further incubated. The medium in the lower compartment was then examined for the presence of MRSA by plating the medium on selective agar plates for MRSA growth (BBL CHROMagar^®^ MRSA, BD). MRSA produces typical pink colonies resulting from hydrolysis of the chromogens added to the agar, while colonies from other bacterial species appear white. Success of each material group was analyzed via Kaplan–Meier-survival analysis, and the average probability of success (event-free time) was determined by calculating the area under the curve (AUC) for each material group.

### 2.7. Quantitative Microleakage 

Quantitative analysis of microleakage through the temporary filling materials was performed using the glucose leakage method by modifying a previously described protocol [17,18]. Seventy-five hollow test specimens were prepared and filled with the temporary filling materials as described above. A setup similar to that employed for the bacterial penetration experiment was used, involving a crimp top vial as a lower compartment and a reaction tube as an upper compartment, with the hollow test specimen containing the temporary filling materials serving as the only interface. A total of 3.5 mL of water was added to the lower compartment, and 1 M D-glucose solution was added to the upper compartment as the tracer substance. Both the water and glucose contained 0.2% sodium azide to prevent bacterial growth. A sterile 25 G × 1 1/2” (0.5 mm × 40 mm) cannula (B.Braun, Melsungen, Germany) was placed in the lower compartment of each specimen to regularly withdraw liquid with a syringe for measurement. The experimental setup is shown in Figure 1b. The specimens were incubated at 37 °C and 100% humidity and in 5.8% CO_2_ for 28 days. Glucose leakage through the temporary filling materials was assessed every 2 days by quantifying the glucose concentration in the lower compartment via an enzymatic assay (Glucose (HK) Assay Kit, Merck, Darmstadt, Germany). The Glucose (HK) Assay Reagent was reconstituted according to the manufacturer’s instructions, and 200 µL of the test solution was removed from the lower compartment. A total of 100 µL of the detection reagent and 10 µL of the test solution were combined in the wells of a 96-well plate and incubated for 15 min at 37 °C. Absorbance was measured at 340 nm using a spectrophotometer (Varioskan Microplate Reader, Thermo Fisher Scientific, Waltham, MA, USA). To convert the optical densities (OD) to glucose concentrations, a standard curve describing the relationship between OD and predetermined glucose concentrations was generated on each day of measurement.

### 2.8. Statistical Analyses

All statistical evaluations were performed in Python 3.8.0 using the packages *pandas, sklearn*, and *lifelines* for inferential statistics and *matplotlib* for the descriptive analyses [19]. To analyze the probability of success of each material group, a Kaplan–Meier-survival analysis was conducted using the timeframe from the moment the specimens were incubated until the event of interest (i.e., the point at which the medium became turbid) occurred. There were no censored observations during the experiment.

## 3. Results

### 3.1. Minimum Inhibitory Concentrations

The MICs of AMP and CIP for each strain are summarized in Table 2. Both *S. mutans* and *A. actinomycetemcomitans* were susceptible to AMP and CIP according to the breakpoints reported in the CLSI guidelines. MRSA was resistant to AMP and CIP and showed no inhibition zone with respect to either antibiotic. Sample images of agar plates after incubation with the ETEST strips are shown in the Supplementary Material (Figure S2). Based on these results, a concentration of 10 µg/mL was chosen as the amount at which both AMP and CIP would be added to the BHI. No bacterial growth of *S. mutans* and *A. actinomycetemcomitans* was observed after plating and culturing the suspensions grown in AMP/CIP-BHI, and there was no difference in the bacterial growth of the MRSA strains compared to antibiotic-free BHI.

### 3.2. Bacterial Penetration

**Figure 2:**
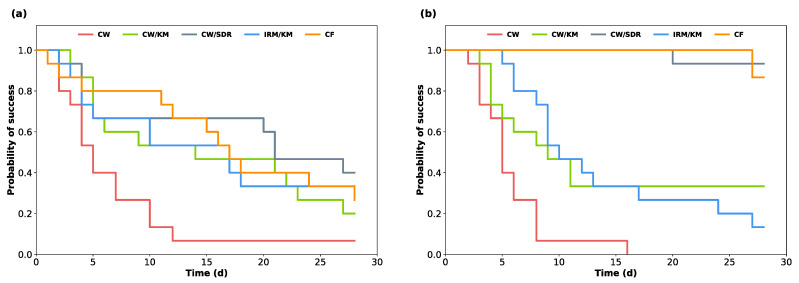
Kaplan–Meier survival analysis indicating the occurrence of bacterial penetration in temporized (**a**) human-extracted teeth and (**b**) 3D-printed hollow test specimens. No observations were censored during the experiment.

Figure 2 shows the Kaplan–Meier survival analysis of the teeth (a) and hollow test specimens (b) filled with the different restorative materials. The average probability of success (event-free time), which was determined by calculating the area under the curve (AUC), and the percentage of event-free specimens after 14 and 28 days of incubation are shown in Table 3 for each material group. In both experimental setups, the highest average event-free time was recorded in the CW/SDR and CF groups, whereas CW alone had the smallest AUC. The largest differences between the AUC obtained from the setup with the hollow test specimens and the setup with teeth was observed for the CW/SDR and CF groups. CW/KM and IRM/KM showed a similar success rate in both experimental setups. After 14 days of incubation, CW/SDR and CF had the highest percentages of event-free specimens in both experimental setups. At the end of the observation period (28 days), the highest number of tight specimens was observed in the specimens sealed with CW/SDR in both setups.

### 3.3. Glucose Leakage

**Figure 3:**
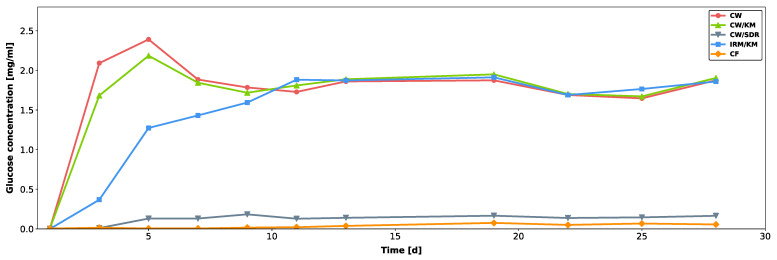
Glucose microleakage of temporized 3D-printed hollow test specimens.

The results of the glucose leakage experiment are shown in Figure 3. In the CW, CW/KM, and IRM/KM groups, the glucose concentration in the lower compartment increased rapidly within the first five days. After 10 days of incubation, the glucose levels in the lower compartment were saturated in these three groups. The lowest values of glucose leakage were observed in the specimens sealed with CF and CW/SDR, where the glucose levels in the lower compartment did not exceed concentrations of 0.07 mg/mL and 0.18 mg/mL, respectively. 

## 4. Discussion

Temporary restorations are a potential weak spot of root canal treatments because bacteria and oral fluids may penetrate along the margins of seemingly intact restorations, leaving clinicians uncertain about the sterility of the pulp chamber and the root canals after temporization. To abolish the need for dressing agents and a temporary seal, one-visit treatments have been suggested, particularly for clinical cases with necrotic pulps or vital teeth with clinical signs of inflammation [20]. Although one-visit treatments have been proven to be just as effective as multi-visit treatments in terms of clinical success, they are associated with a higher risk of postoperative pain or flare-ups [20,21]. Therefore, a multi-visit treatment protocol is the preferable alternative for infected teeth causing preoperative pain in order to reduce the chances for postoperative complications as well as the unnecessary or excessive use of nonsteroidal anti-inflammatory drugs, which are commonly used to treat postoperative endodontic pain [20,22,23]. 

During multi-visit treatments, a sufficient temporary coronal seal is a prerequisite for avoiding the contamination of the root canal system with oral fluids and bacteria. The presence of bacteria in the root canal at the time of filling is considered a risk factor for developing posttreatment apical periodontitis [24]. Ideally, the root canal system will be thoroughly disinfected at the final appointment to remove any residual bacteria. Nonetheless, leakage through the temporary restoration may facilitate entry of *Enterococcus faecalis*, a bacterial species that is typically found in teeth in need of endodontic retreatment and, less frequently, in primary endodontic infections [25,26,27]. The problem with this species is that it is able to withstand conventional eradication measures because it is equipped with certain survival and virulence factors, such as the ability to invade dentinal tubules, survive extremely alkaline environments, and resist nutritional deprivation [28]. *E. faecalis* and several other bacteria found in persistent infections have been demonstrated to be resistant to calcium hydroxide dressing agents, which highlights the necessity of avoiding the colonization of the root canal system with such bacteria by ensuring a tight coronal seal [29].

In this study, bacterial and glucose leakage was assessed over an observation period of 28 days to judge the sealing ability of the temporary filling materials as a function of time. We employed two methods to address different factors relating to the tightness of the restorations. While bacterial penetration seems the most plausible and biologically relevant method for our research question, it does not account for voids that are smaller than the average size of a bacterial cell (0.5–1.0 µm). However, these gaps are of interest from a clinical point of view because they may provide pathways for smaller molecules, such as toxins and other bacterial products, to travel to the periapex [30]. Several methods for assessing microleakage have been previously described. Most methods are based on the same principle, that is, detecting a tracer upon penetration along the margins of the coronal restoration or the obturated canal of an extracted tooth. The most frequently used method is dye penetration with dyes such as methylene blue, basic fuchsin, or India ink. However, the problem with this method is that it leads to a great deal of variation in the results because it is usually associated with assigning a score using a scoring system, which leads to a subjective interpretation of the degree of leakage [31]. Furthermore, it has been reported that dye penetration can lead to misleading conclusions due to alterations of the properties of the dye depending on the pH or the presence of ions [32]. The use of radioactive isotopes as tracers allows for better quantification of the microleakage via the placement of a known quantity of an isotope into the pulp chamber of an extracted tooth and allowing the tracer to leak into an outside medium, where the amount of the radioactive isotope can be measured using a scintillation counter [33]. However, the downsides of this method are that it is extremely technique-sensitive and has questionable clinical relevance in addition to posing a risk due to the exposure to potentially hazardous radiation [31].

The glucose leakage model modified according to Xu et al. allows for a nondestructive quantification of microleakage over time without suffering from the shortcomings of the methods mentioned above [17]. Glucose seemed to be a suitable tracer for our research question, not only because it serves as an indicator of smaller gaps within a restoration due to its small molecular size (180 Da) but also because glucose serves as a nutrient for bacteria, which, once it has penetrated through the temporary restoration, may promote the growth of residual bacteria colonizing the root canal. The advantages of this method are its great sensitivity, ease of operation, and comparatively low costs. The fact that glucose is relatively stable in vitro is a further advantage of the glucose penetration method. This feature allows us to determine microleakage continuously, whereas other methods such as dye penetration require an end-point for assessing the penetration depth along the root canal.

Previous studies employing bacteria as a tracer for leakage used species that are often found in infected root canals, such as *Enterococcus faecalis*, *Streptococcus mutans*, or *Streptococcus gordonii* [8,12,34]. Using bacteria in accordance with their clinical relevance seems to be a viable approach; however, this protocol is fraught with difficulties in controlling the bacterial populations due to potential cross-contamination and other bacteria originating from the extracted teeth. To overcome this common limitation, we modified the methodology in our study and used MRSA as a microbial tracer in combination with a culture medium containing antibiotics. One may now question the clinical relevance of this approach, as MRSA is not a typical species found in root canal biofilms [35]. However, this concept is based on the premise that bacteria do not significantly vary in size and that our goal is to make a dichotomous decision on whether bacterial cells were able to penetrate along the margins of the restoration. The advantage of this method is that it allows for the selective growth of the tracer bacteria while avoiding contamination with other bacterial species. To determine suitable antibiotic concentrations for a BHI medium with efficient antibacterial activity against Gram-positive and Gram-negative bacteria, *S. mutans* and *A. actinomycetemcomitans* were used. Aside from these two species, which served as representative bacteria for laboratory cross-contamination, *S. mutans* and *A. actinomycetemcomitans* were chosen because they are typical species found in teeth affected by the most common oral diseases, i.e., caries and periodontitis. In addition, sterility was double-checked upon the occurrence of an event by plating the medium from the lower compartment on chromogenic agar plates that allow for the selective identification of MRSA growth. 

The results from the bacterial leakage experiments in conjunction with those from the glucose penetration experiment may reflect the sealing ability of the tested materials in a clinical context. Our proposition that the presence of nutrients in the root canal and bacteria may be correlated was confirmed by the fact that the specimens with a high degree of glucose leakage also had a high degree of bacterial leakage. This was the case for the access cavities sealed with CW, CW/KM, or IRM/KM. In the CW and CW/KM groups, we observed a drop in glucose concentrations between days 5 and 7. This reduction might have been to possible interactions between glucose and components within the restorative materials. Previous research has demonstrated that certain endodontic filling materials, especially those containing calcium hydroxide, react with glucose to form gluconic acid, thereby reducing the glucose concentration. A further compound the authors tested with regard to its reactivity with glucose was calcium sulphate, which also led to a slight reduction in glucose content, but the difference in concentrations was not significant [36]. Based on this information, calcium sulphate, one of the main components of Cavit, may have caused the glucose concentration to slightly drop in the CW and CW/KM specimens, especially since these restorations showed early signs of microleakage, allowing for the glucose in the lower compartment to interact with the CW portion. Furthermore, the authors of the previously mentioned study had chosen a period of 7 days for the immersion of the specimens in the glucose solution, which happens to be the same time until the glucose concentration dropped in the presence of the CW and CW/KM specimens.

Based on the findings obtained from several studies examining the adhesive bond to the tooth, it is not surprising that specimens sealed with CW/SDR were superior in terms of tightness [37]. The fact that no drop in glucose concentration occurred in the CW/SDR specimens supports this proposition, as the glucose probably never reached the CW portion of CW/SDR in significant concentrations. Access cavities that were sealed solely with CW presented the highest levels of bacterial leakage, whereas CW with a layer of KM on top seemed to provide a slightly improved seal against bacterial leakage but not against glucose microleakage. IRM/KM and CW/KM presented similar results in terms of bacterial and glucose leakage; however, the onset of glucose leakage from the IRM/KM specimens occurred later than that from the CW/KM specimens. These findings are in accordance with previous studies that demonstrated an equal or better seal using IRM than that provided by Cavit against bacterial penetration [12,38,39]. We obtained promising results for CF, a light-curing methacrylate-based material, which has not yet been as widely studied as Cavit and IRM. Previous studies that assessed the marginal tightness of different restorative materials with dye penetration (fluorescent silver staining and methylene blue) demonstrated an equal or superior seal using CF compared to that using IRM or Cavit G [10,40]. A further study examined the sealing ability of temporary restorations in previously restored teeth (for which dye penetration with basic fuchsine was employed) and found that Clip, the forerunner material of CF, provided the best seal against microleakage not only at amalgam and composite interfaces but also at the tooth–tissue interface [41]. The only other in vitro study employing bacteria as a tracer for coronal leakage reported a 50% chance of success for teeth sealed with Clip after an incubation time of 30 days with a *S. mutans* bacterial suspension [42]. The good sealing ability of CF may be attributed to its expansion properties reported by the manufacturer, which have not yet been confirmed through independent research. The product data sheet for CF indicates a 0.5% level of water sorption after immersion in water for 14 days, which could have contributed to the tight seal within the access cavities. Moreover, it should be noted that CF, a material with no known mechanism of adhesive bonding to tooth tissues, has been reported to provide an excellent bond with other methacrylate-based materials [41,43]. This feature must be considered when interpreting the results from our experiment with the 3D-printed hollow test specimens to avoid an overestimation of the material’s sealing ability. Even without pretreatment of the hollow test specimens, the unpolymerized methacrylates in CF might have created a chemical bond with the methacrylates in the resin, which may explain the low occurrence of events in the setup with the 3D-printed test specimens. This notion also applies to the 3D-printed specimens sealed with CW/SDR, which was used along with a universal adhesive capable of creating a chemical bond with the resin’s surface. In contrast, the properties of the 3D-printed forms might have led to an underestimation of the tightness of CW/KM and IRM/KM because KM is a GIC, which is primarily designed to bond to tooth tissues and not methacrylates. On the other hand, CW and IRM do not have an adhesive bonding mechanism. Instead, their sealing ability is based on hygroscopic expansion, which produces a seal within the cavity [44]. It is unlikely that the sealing ability of CW and IRM was impaired by the use of the 3D-printed hollow test specimens, especially since a similar survival curve was obtained for both experimental setups. However, it should be noted that teeth in need of root canal treatment often have coronal restorations that are not always removed prior to gaining access to the pulp chamber. This may significantly affect the sealing properties of the materials depending on the type of material used for the coronal restoration. Therefore, CF may be an option when sealing the access cavities of teeth with coronal composite restorations, or for methacrylate-based materials in general. From a practical point of view, a disadvantage of using materials with a dentin-bonding agent is that they are harder to remove than cements such as CW or IRM. As re-entry through composite restorations requires the use of diamond burs, there is a higher risk of weakening the tooth when attempting to remove all adhesive residues potentially blocking the dentin tubules of the pulp chamber wall. The double-layered restorations involving a coronal layer of composite or GIC require a clean dentin surface void of CW or IRM residues for a sufficient seal. In particular, IRM residues in the dentin tubules have been reported to affect the microtensile bond strength of adhesives because IRM contains eugenol, which acts as a radical scavenger and inhibits the polymerization process of methacrylates [45,46]. CF, on the other hand, does not present any type of dentin adhesion and is thus comparatively easy to remove in bulk. Even if the material is impacted in retentive cavities, the removal of CF only requires one sectioning procedure along the center of the restoration.

It should be mentioned that the significance of our results is limited by the in vitro nature of this study, which does not account for the complex environment in the human oral cavity. Since the extracted teeth were not subjected to masticatory forces, our results do not include potential failures due to loss of marginal integrity, which may severely affect the tightness of the restorations. The integrity of the restorative materials may also be influenced by patient-specific factors, such as bruxism, oral hygiene, or dietary habits inducing recurrent pH drops in the oral cavity. Although temporary filling materials only remain in the access cavity for a short time, the loss of tightness induced by such conditions may affect the sterility of the pulp chamber and hence endodontic success. Therefore, further microleakage studies simulating different oral conditions in addition to in vivo studies are necessary to confirm our results. 

## 5. Conclusions

Based on our in vitro results, CW/SDR and CF provided the best results in terms of coronal tightness. There are implications that CF or CW/SDR (used with a self-adhesive dentin bonding agent) should be favored when sealing access cavities of previously restored teeth, especially when the coronal restoration contains methacrylate components. Regarding double-layer techniques, there seemed to be no differences in outcome between the CW/KM and IRM/KM specimens. From a methodological point of view, our study demonstrates that the assessment of bacterial penetration with MRSA as a tracer combined with the evaluation of glucose leakage provides reliable results for judging the sealing ability of restorative materials.

## Figures and Tables

**Table 1:** Materials, chemical composition, and application procedures of the materials used in this study.

Material	Manufacturer	LOT Number	Material Class	Application Procedure
Cavit W (CW)	3M (St. Paul, MN, USA)	618059	Eugenol-free calcium sulphate-zinc oxide-based cement	Apply a 5 mm layer (3 mm for double-layer restorations) to cavity, add moisture to cavity
Ketac Molar Aplicap (KM)	3M	624122	Glass ionomer cement	Activate capsule for 2 s, mix at 4300 rpm for 10 s, apply a 2 mm layer to cavity
Smart Dentin Replacement flow+ (SDR)	Dentsply Sirona (York, PA, USA)	1604000968	Flowable bulk fill composite	Apply adhesive (Scotchbond Universal) to cavity, air dry for 5 s, light cure for 20 s, apply a 3 mm layer of SDR to cavity, light-cure for 20 s
Intermediate restorative material (IRM)	Dentsply Sirona	1606000692	Zinc oxide eugenol cement	Mix powder and liquid, apply a 3 mm layer to cavity
Clip F (CF)	Voco Dental GmbH (Cuxhaven, Germany)	1615395	Fluoride-releasing, light-curing restorative material	Apply a 5 mm thick layer to cavity, light cure for 40 s

**Table 2:** Minimum inhibitory concentrations (MIC) of bacterial strains against Ampicillin and Ciprofloxaicin determined using the ETEST method. MICs interpreted according to guidelines of the Clinical and Laboratory Standards Institute (CLSI). R, resistant; S, susceptible.

Strain	Antibiotic	Range	MIC (µg/mL)	Interpretation
*S. mutans*	Ampicillin	0.016–256	0.094	S
	Ciprofloxacin	0.002–32	1.00	S
*A. actinomycetemcomitans*	Ampicillin	0.016–256	1.00	S
	Ciprofloxacin	0.002–32	0.004	S
MRSA	Ampicillin	0.016–256	>256	R
	Ciprofloxacin	0.002–32	>32	R

**Table 3:** Area under the Kaplan–Meier curve (AUC) indicating the average probability of success (event-free time) for each material group along with the percentage of event-free specimens after 14 and 28 days of incubation. All values are shown for the experimental setups with extracted teeth and hollow test specimens.

	Cavit W	Cavit W + Ketac Molar	Cavit W + SDR	IRM + Ketac Molar	Clip F
AUC Teeth	6.20	14.00	17.20	14.30	16.40
Success after 14 days (%)	6.67	46.67	66.67	53.33	66.67
Success after 28 days (%)	6.67	20.00	40.00	33.33	26.67
AUC Hollow test specimens	4.93	13.17	26.80	13.07	26.07
Success after 14 days (%)	6.67	33.33	100.00	33.33	100.00
Success after 28 days (%)	0.00	33.33	93.33	13.33	86.67

## Data Availability

Publicly available datasets were analyzed in this study. These data can be found here: Würsching: Sabina Noreen und Moser, Luise und Obermeier, Katharina Theresa und Kollmuß, Maximilian: Microleakage and economic efficiency of restorative materials used for temporization of endodontic access cavities. Open Data LMU https://doi.org/10.5282/ubm/data.382, accessed on 23 May 2023.

## Supplementary Materials

The following supporting information can be downloaded at: https://www.mdpi.com/article/10.3390/jcm12144762/s1, Figure S1: Dimensions of the conical cylinder used for standardized hollow test specimens; Figure S2: Sample agar plates showing the minimum inhibitory concentrations of ampicillin and ciprofloxacin.
