# Supplementary material for: Microleakage of Restorative Materials Used for Temporization of Endodontic Access Cavities
Source: J Clin Med. 2023 Jul 18;12(14):4762. doi: 10.3390/jcm12144762 (PMC10381707; doi:10.3390/jcm12144762)

S2. Sample agar plates showing the minimum inhibitory concentrations of ampicillin and ciprofloxacin

a) MIC of ampicillin and ciprofloxacin against *S. mutans*:

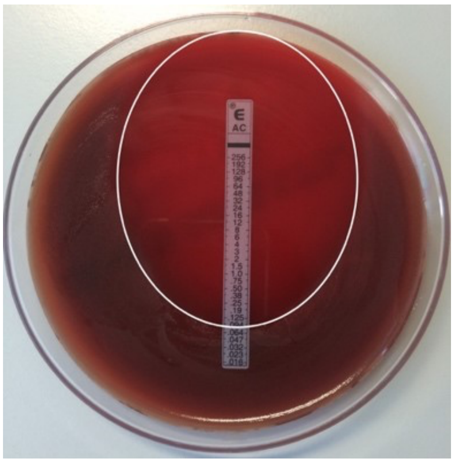

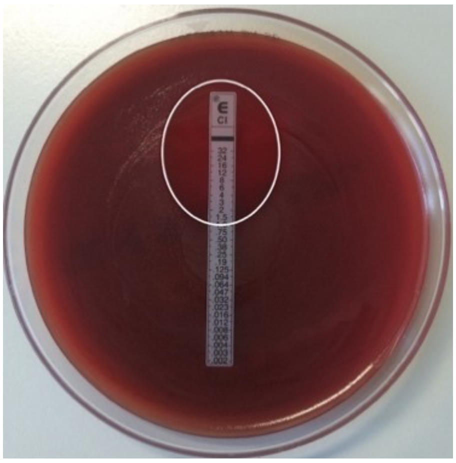

b) MIC of ampicillin and ciprofloxacin against *A. actinomycetemcomitans*:

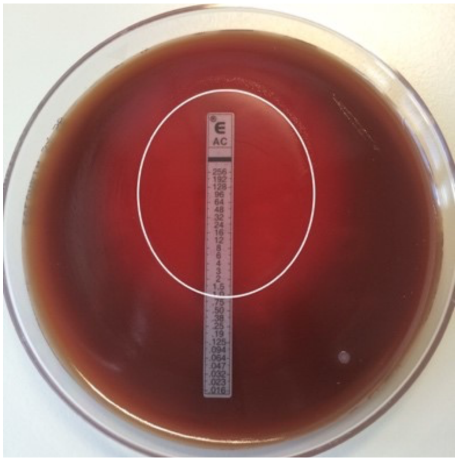

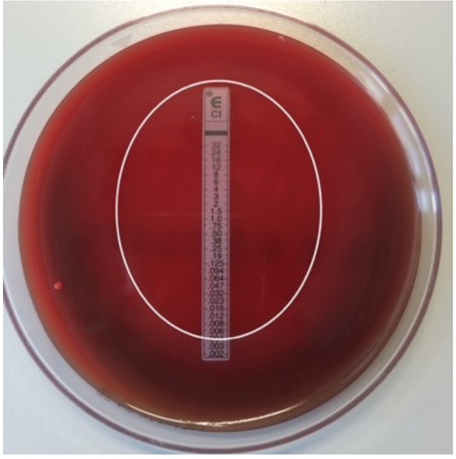

c) MIC of ampicillin and ciprofloxacin against *MRSA*:

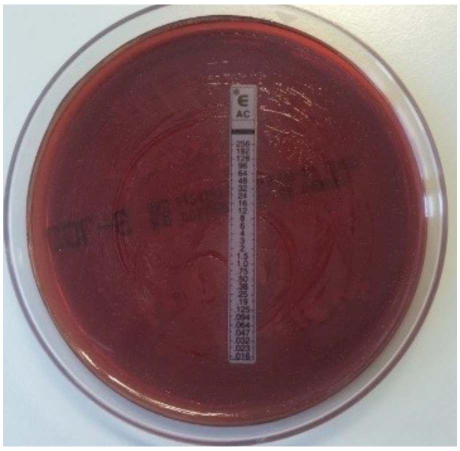

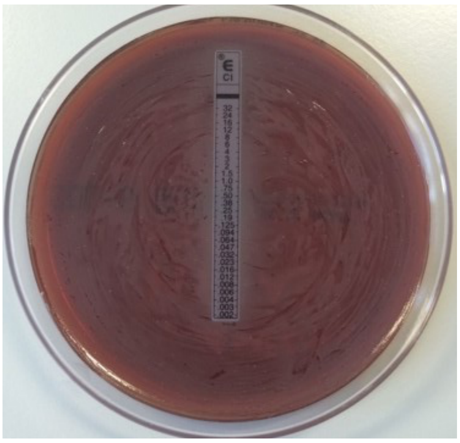

Supplement: Supplementary file 1 [file jcm-12-04762-s001.zip › Figure S2.pdf]
