# Supplementary figures and images for: Microleakage of Restorative Materials Used for Temporization of Endodontic Access Cavities
Source: J Clin Med. 2023 Jul 18;12(14):4762. doi: 10.3390/jcm12144762 (PMC10381707; doi:10.3390/jcm12144762)

**S1.** Dimensions of the conical cylinder used for standardized access openings

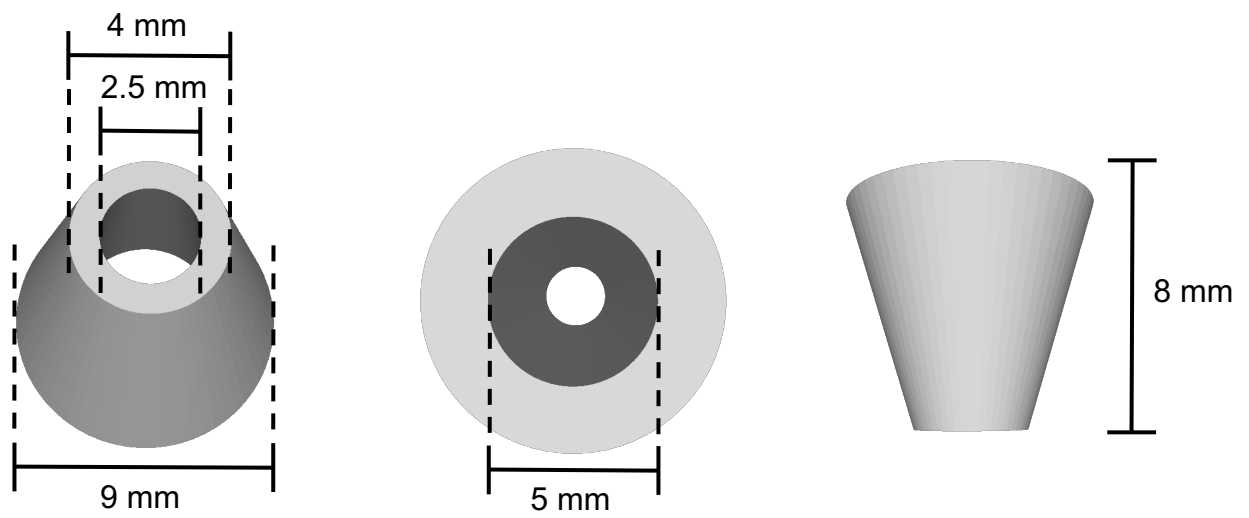

Supplement: Supplementary file 1 [file jcm-12-04762-s001.zip › Figure S1.pdf]
